# Case Report: pharmaceutical care in a case of complicated urinary tract infection combined with disseminated *Nocardia brasiliensis* infection

**DOI:** 10.3389/fmed.2026.1839868

**Published:** 2026-06-11

**Authors:** Houjun Pang, Chuan Pi, Pengyu Shen, Ziru Tang, Erhao Bao, Xing Luo, Qixiong Zhang

**Affiliations:** 1 Department of Pharmacy, Sichuan Provincial People’s Hospital East Sichuan Hospital & Dazhou First People’s Hospital (Dazhou Maternal and Child Health Hospital), Dazhou, China; 2 Department of Rheumatology and Immunology, Sichuan Provincial People’s Hospital East Sichuan Hospital & Dazhou First People’s Hospital, Dazhou, China; 3 Department of Urology, Sichuan Provincial People’s Hospital East Sichuan Hospital & Dazhou First People’s Hospital, Dazhou, China; 4 Department of Pharmacy, Sichuan Provincial People’s Hospital, Chengdu, China

**Keywords:** antimicrobial agents, complicated urinary tract infection, disseminated infection, nocardiosis, pharmaceutical care

## Abstract

Given the increasing prevalence of multidrug-resistant opportunistic pathogens and the high mortality rate associated with delayed diagnosis of disseminated infections, there is an urgent need for rapid diagnostic tools and closely monitored, individualized anti-infective strategies. This study aimed to explore the critical role of comprehensive pharmaceutical care in managing disseminated *Nocardia* infections complicated by complicated urinary tract infection (cUTI). Through detailed documentation of a 67-year-old male patient, this study focuses on optimizing antimicrobial regimens based on pathogenetic findings and adjusting treatments for severe adverse reactions. The patient was diagnosed with disseminated *Nocardia brasiliensis* infection complicated by *Enterococcus faecalis* urinary tract infection using metagenomic next-generation sequencing (mNGS). The treatment process underwent two critical adjustments. First, during the efficacy optimization phase, the initial empirical meropenem therapy was modified to a reinforced regimen centered on trimethoprim-sulfamethoxazole (TMP-SMX), combined with linezolid and short-term amikacin, effectively controlling the spread of infection. Subsequently, during the safety optimization phase, the patient developed severe thrombocytopenia during sequential oral therapy. Prompt identification and switching to amoxicillin/clavulanate potassium resolved the adverse reactions, enabling successful continuation of subsequent treatment. Follow-up revealed a favorable patient recovery. This case demonstrates that for such complex mixed infections, rapid pathogen diagnosis represented by mNGS serves as the starting point for precision treatment, whereas the intensive combination regimen centered on TMP-SMX forms the foundation for controlling disseminated *Nocardia* infection. More importantly, the core insight from this case is that successful treatment relies not only on appropriate initial medication, but more critically, on proactive, dynamic pharmaceutical monitoring throughout long-term therapy. This enables early intervention for severe adverse drug reactions and timely, flexible adjustments to treatment regimens, which are essential components for ensuring ultimate therapeutic success in patients with such complex infections.

## 1 Case information

A 67-year-old male patient presented with complaints of “urinary frequency and dysuria for over 20 days, accompanied by limb pain and rash for 5 days.” Twenty days prior, the patient developed urinary frequency and dysuria without an apparent cause, along with urinary hesitancy and incomplete voiding. He experienced no fever, chills, back pain, or hematuria, and did not seek medical attention. Over 10 days ago, the symptoms had worsened with the onset of dysuria. He sought care at a local hospital where he was diagnosed with “urinary tract infection and acute prostatitis.” Treatment included cefoperazone/sulbactam for infection and placement of an indwelling urinary catheter, which resulted in slight symptom relief. However, 5 days ago, the patient’s condition changed, with sudden swelling and pain in the left foot, accompanied by increased local skin temperature. Simultaneously, scattered red, tender nodular-like rashes appeared on both the lower limbs. Symptoms did not improve after symptomatic treatment at the external hospital (specific medications were unknown). The patient was transferred to our hospital on May 15, 2025, for further evaluation and treatment.

On admission, physical examination revealed a temperature of 36.5 °C, pulse rate of 90 beats/min, respiratory rate of 19 breaths/min, and blood pressure of 146/98 mm Hg.

Complete blood count: WBC 14.84 × 10^9^/L (neutrophils 82%), Hb 81 g/L, PTL 488 × 10^9^/L. Urinalysis: WBC 1344/μL, microscopic WBC 336/HP. Inflammatory markers: ESR, 87 mm/H, hs-CRP 128.10 mg/L; random blood glucose 11.21 mmol/L. Abdominal CT: blurred margins of the bladder, prostate, and rectum; diffuse thickening of the pelvic floor fascia. Urinary system ultrasound: enlarged prostate (5.7 cm × 5.6 cm × 5.3 cm).

**FIGURE 1:**
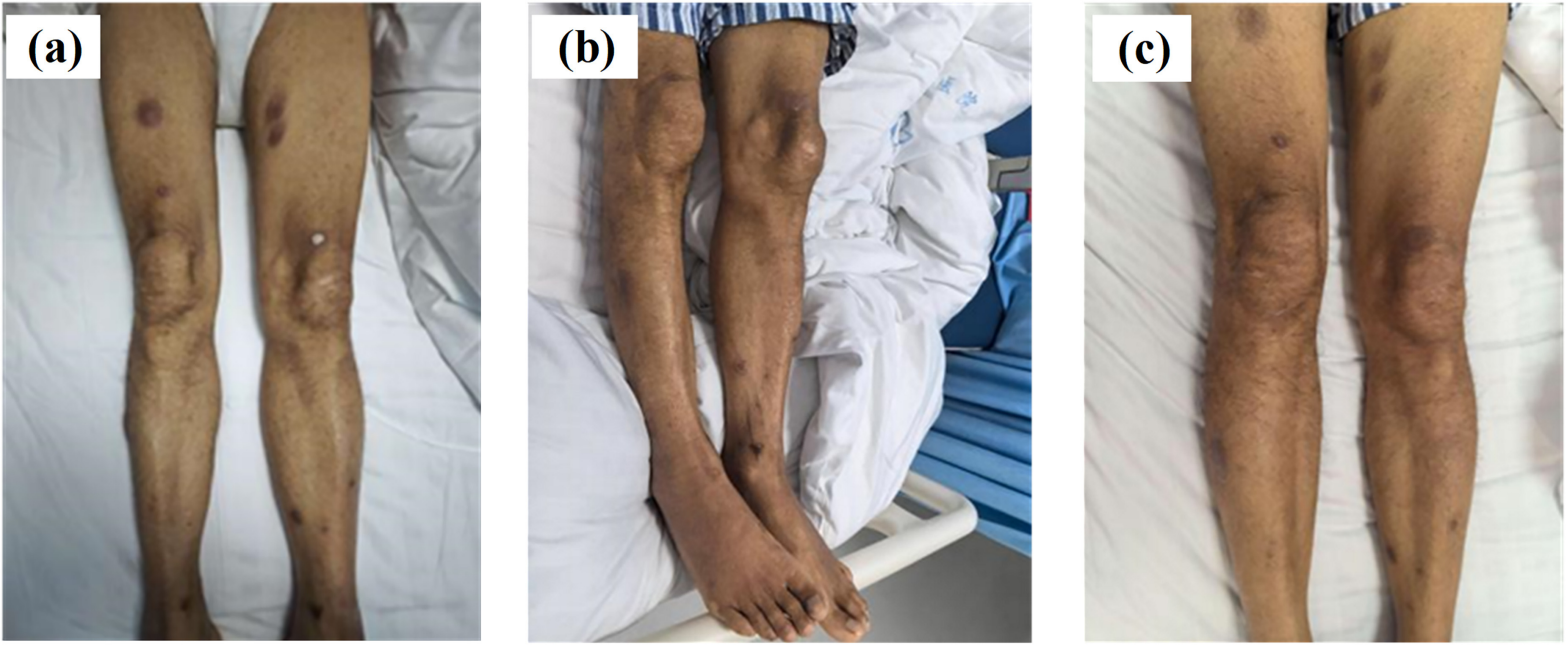
**(a)** Pus spots and nodules on skin of both lower limbs at admission. **(b)** Reduced rash on both lower limbs. **(c)** Skin lesions absorption in both lower limbc.

**FIGURE 2:**
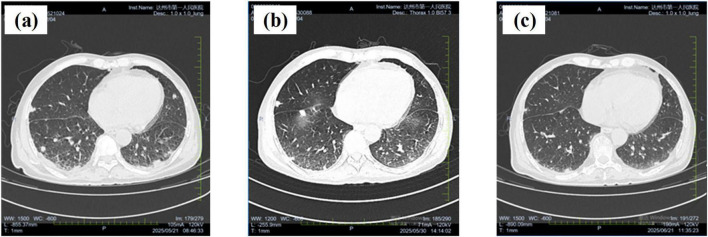
**(a)** Chest CT on 2025-05-21. **(b)** Chest CT on 2025-05-30. **(c)** Chest CT on 2025-06-21.

Therapeutic approach: Empirical antimicrobial therapy with meropenem 1 g q8h was initiated, but the patient continued to experience intermittent fever, with peak temperatures reaching 39°C. Some skin nodules developed into pustules (Figure 1a), indicating inadequate infection control. Further pathogen testing was conducted: Blood mNGS on May 18, 2025 (with results reported within 24 h of sample collection), identified *Nocardia brasiliensis* (1498 sequence reads); urine culture on May 19, 2025, detected *Enterococcus faecalis* (10^5^ CFU/ml); and bacterial culture of skin abscess drainage also yielded *Nocardia brasiliensis*. Based on these findings, antimicrobial therapy was adjusted to linezolid (0.6 g q12h) combined with trimethoprim-sulfamethoxazole (SMZ-TMP 0.8 g tid). During this period, the patient’s cough and sputum production worsened. A chest CT scan on May 21, 2025, revealed multiple nodules with cavitation in both lungs (Figure 2a), consistent with the radiographic features of disseminated *Nocardia* infection. Owing to progressive pulmonary lesions and worsening symptoms, amikacin (0.4 g qd) was added to enhance antimicrobial coverage. Approximately 2 weeks after initiating intravenous therapy, the patient’s temperature gradually normalized, skin abscesses partially dried, and respiratory symptoms markedly improved. The treatment was switched to oral sequential therapy with linezolid 0.6 g bid combined with SMZ-TMP 0.96 g tid.

However, 30 days into treatment (June 17, 2025), follow-up blood tests revealed a precipitous drop in platelets to 72 × 10^9^/L and progressive decrease in hemoglobin to 50 g/L, suggesting linezolid and SMZ-TMP-related bone marrow suppression. Both drugs were discontinued immediately. Platelet-stimulating agents were administered for symptomatic support, and the antinocardial regimen was switched to oral amoxicillin-clavulanate potassium. Following the adjustment, platelet counts gradually recovered, bleeding risk resolved, and skin and pulmonary infections improved further.

Initial presentation (May 21, 2025, Figure 2a): Chest computed tomography (CT) revealed multiple bilateral pulmonary nodules with partial cavitation, suggestive of acute disseminated infection. Multiple erythematous nodules were visible on the lower extremities, and some were ulcerated with purulent foci (Figure 1a).

Mid-treatment phase (May 30, 2025; Figure 2b): Pulmonary lesions decreased in size, with clearer cavity boundaries, suggesting that the antimicrobial regimen was effective. The lower limb rash improved, and some pustules had crusted (Figure 1b).

Consolidation phase (June 21, 2025; Figure 2c): Chest CT showed a significant reduction in nodules, thinning of the cavity walls, and absorption of inflammatory exudates. Skin lesions on both lower limbs were mostly resolved, leaving only pigmentation- and scar-like changes (Figure 1c).

**FIGURE 3:**
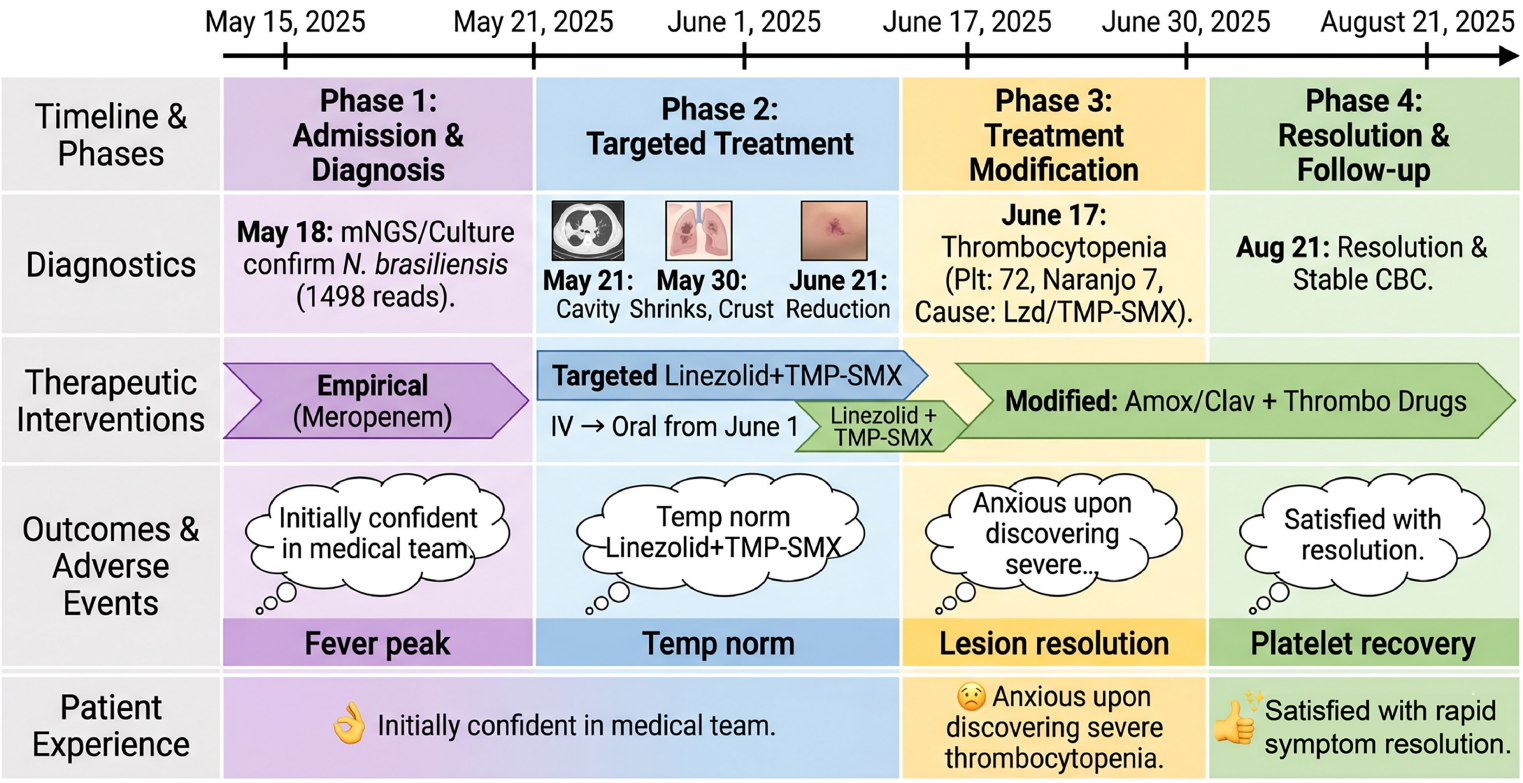
Timeline of the patient’s episode of care, illustrating diagnostic milestones, therapeutic interventions, and clinical outcomes.

At discharge, the patient’s vital signs were stable with no fever. The skin nodules were nearly resolved, and the cough and sputum production were significantly reduced. The patient was discharged with oral amoxicillin/clavulanate for continued antinocardial therapy, with regular monitoring of complete blood count, liver and kidney function, and imaging follow-up. After 2 months of follow-up, platelet and hemoglobin levels remained stable, urinary symptoms disappeared, and pulmonary lesions showed further absorption, indicating effective treatment. The patient is currently under follow-up. The detailed timeline of the patient’s clinical course, including diagnostic milestones and therapeutic transitions, is summarized in Figure 3.

## 2 Patient perspective

Throughout the treatment process, the patient remained highly cooperative and expressed significant trust in the clinical pharmacy and medical team. When severe thrombocytopenia occurred, the patient felt anxious initially; however, after the pharmacist explained the drug-related mechanism and the rationale for the regimen switch, the patient’s concerns were alleviated. At the two-month follow-up, the patient reported feeling physically restored and was satisfied with the rapid resolution of his complex symptoms without permanent complications.

## 3 Discussion

### 3.1 Disseminated nocardiosis

Disseminated nocardiosis is a rare but severe infectious disease caused by *Nocardia* species and is commonly seen in immunocompromised patients (1, 2). Although this 67-year-old male patient did not have a typical immunodeficiency, his underlying diabetes, long-term indwelling urinary catheter, and recent history of broad-spectrum antibiotic use collectively created a high-risk background for infection. Nocardial bacteria typically enter the human body through direct contact or inhalation, causing localized or systemic infections. The skin and lungs are the most commonly affected organs, whereas brain abscesses represent a severe complication, often secondary to infection foci elsewhere in the body (2, 3). However, the route of infection and dissemination pattern in this case were markedly atypical; the initial presentation was a complicated urinary tract infection, followed by a skin abscess, and ultimately hematogenous spread to the lungs, forming multiple cavitary nodules, while the central nervous system was spared. This “urinary tract-skin-lung” dissemination pathway is highly prone to misdiagnosis in the early stages, underscoring the critical importance of maintaining vigilance for atypical infections and conducting precise pathogen identification.

Diagnosing nocardiosis is challenging because of its clinical presentation, often mimicking other diseases such as lung cancer, tuberculosis, or fungal infections. Imaging studies and microbial cultures are key diagnostic tools; however, the slow growth of *Nocardia* species can lead to diagnostic delays. The complexity of this case further stems from the presence of mixed infections and an atypical dissemination pattern. Empirical treatment (cefoperazone-sulbactam and meropenem) proved ineffective, indicating the need to move beyond conventional thinking. Metagenomic next-generation sequencing (mNGS) has proven pivotal, rapid, and unbiased in identifying *Nocardia brasiliensis*, providing decisive guidance for subsequent therapy. Dynamic imaging studies further confirmed the disseminated infection.

Treatment complexity primarily stems from the significant differences in antimicrobial susceptibility among *Nocardia* species. The literature indicates that *Nocardia brasiliensis* typically exhibits sensitivity to sulfonamides (e.g., sulfamethoxazole-trimethoprim, SMZ-TMP) but high resistance to carbapenems (4–6), directly explaining the poor efficacy of the initial meropenem therapy in this case. Therefore, obtaining early pathogen evidence is essential for achieving precise treatment. Throughout therapy, close integration of clinical symptoms, dynamic imaging changes, and adverse drug reactions (such as the subsequent bone marrow suppression observed in this case) is required for continuous monitoring and individualized adjustments to ensure treatment efficacy and safety.

### 3.2 Complicated urinary tract infection

Complicated urinary tract infection (cUTI) is a common infectious disease in clinical practice, and its diagnosis and treatment are often more challenging owing to concomitant anatomical or functional abnormalities of the urinary system (7). As a gateway for mixed infections and a diagnostic/therapeutic challenge, this patient presented with multiple cUTI risk factors: diabetes, significant prostatic hyperplasia, and long-term indwelling urinary catheterization. These factors collectively lead to altered urinary dynamics and compromised local mucosal defense barriers (objectively evidenced by symptoms of severe dysuria, microscopic hematuria, and imaging findings of diffuse pelvic fascia thickening). These compromised barriers created an optimal portal of entry for pathogen colonization and ascending infection (8), forming the initial pathological basis for this mixed infection.

The microbiological findings in this case demonstrated how cUTI can evolve into more complex systemic infections under specific circumstances. Urine culture clearly identified *Enterococcus faecalis* (105 CFU/ml), whereas blood mNGS confirmed disseminated *Nocardia brasiliensis*. It is highly probable that the impaired urothelium served as the primary entry point for *N. brasiliensis*, allowing it to invade the bloodstream and subsequently disseminate to the skin and lungs. This mixed infection pattern involving “a resistant Gram-positive bacterium (*Enterococcus faecalis*) and a rare opportunistic pathogen (*Nocardia brasiliensis*)” presents significant challenges: *Enterococcus faecalis* commonly exhibits resistance to most routine urinary tract antimicrobials, retaining susceptibility only to a limited number of agents such as vancomycin and linezolid; while *Nocardia brasiliensis* exhibits inherent high resistance to β-lactam antibiotics. This dual-resistance background explains why initial empirical antimicrobial therapy targeting common pathogens (cefoperazone-sulbactam, meropenem) failed to control the infection, leading to severe dissemination from the urinary tract to the skin and lungs.

Therefore, this case strongly suggests that for cUTI patients with multiple high-risk factors (e.g., diabetes, urinary tract obstruction, long-term catheterization), if initial standard empirical therapy shows poor response or signs of multisystem dissemination (e.g., fever, skin lesions, respiratory symptoms), clinicians must move beyond considering only “common pathogens” and remain highly vigilant for the possibility of mixed infections involving rare or opportunistic pathogens such as *Nocardia*. Comprehensive pathogen testing (including traditional culture and molecular diagnostic techniques, such as mNGS) is crucial for breaking diagnostic impasses and shifting toward precision treatment. This underscores that in complex infection management, cUTI is not merely a localized issue but may represent a systemic infection source. Its diagnosis and treatment require multidisciplinary collaboration among infectious diseases, pharmacies, and imaging specialists to enable a rapid, flexible transition from empirical therapy to personalized, pathogen-evidence-based regimens.

### 3.3 Diagnostic essentials and differential diagnosis

The diagnostic process in this patient highlighted the importance of integrating multiple lines of evidence. First, from a microbiological perspective, mNGS rapidly detected *N. brasiliensis* (1498 reads) in the blood, and bacterial culture of aspirated pus from skin abscesses yielded the same organism, indicating a high likelihood of hematogenous dissemination. Concurrently, urine culture demonstrated *E. faecium* at >105 CFU/mL, indicating a UTI complicated by additional pathogens. This convergent set of microbiological findings enabled the clinical team to establish the fundamental presence of a nocardial infection early. Imaging data were critical throughout the diagnostic course: chest CT on May 21, 2025, revealed multiple pulmonary nodules with cavitation, consistent with typical imaging features of disseminated nocardiosis, and subsequent CT scans on May 30 and June 21 of the same year showed gradual lesion reduction, thinning of the cavity walls, and attenuation of the surrounding inflammatory reaction, findings concordant with the patient’s clinical improvement after anti-infective therapy. Dynamic radiologic improvement corroborated the microbiological results, providing dual assurance for the diagnosis and efficacy assessment.

The clinical course of the patient was consistent with the above evidence. The patient initially had only a urinary tract infection, followed by nodular rash and cutaneous abscesses in both lower limbs; hematogenous dissemination gradually involved the lungs. This temporal trajectory, aligned with microbiological and imaging findings, strengthens the diagnostic confidence for disseminated nocardiosis. In practice, this condition is easily confused with other chronic infections: pulmonary tuberculosis may also present with cavitary lesions but typically progresses more slowly and has a distinct microbiology; actinomycosis can present with chronic abscesses and sinus tracts but most commonly involves the cervicofacial or abdominal regions, and histology often reveals “sulfur granules”; invasive fungal infections such as aspergillosis or cryptococcosis can also manifest with multiple nodules or cavities in immunocompromised hosts, but mycological testing usually differentiates them from *Nocardia*. Thus, in this case, etiologic confirmation by mNGS and conventional culture, typical chest CT features, and temporal concordance of skin and pulmonary lesions collectively excluded the above differential diagnoses and established the final diagnosis of disseminated nocardiosis.

## 4 Treatment and pharmaceutical care

### 4.1 Formulation and optimization of the anti-infective regimen

#### 4.1.1 Antimicrobial selection for nocardiosis

Fever and UpToDate recommend trimethoprim-sulfamethoxazole (SMZ-TMP) as the first-line treatment for nocardiosis, with imipenem, amikacin, or linezolid as adjunctive options. After 3–6 weeks of intravenous therapy, the patient was switched to oral combination therapy. For immunocompetent patients, treatment should continue for 3–6 months or longer, while immunocompromised patients require dual-drug combination therapy for at least one year. Initial empirical treatment with meropenem (1 g q8h) demonstrated limited efficacy, consistent with the low carbapenem susceptibility of *N. brasiliensis* (33.3%). This therapeutic experience underscores the critical importance of comprehensive early pathogen testing in patients with suspected *N. brasiliensis* infections. Following the rapid identification of *Nocardia brasiliensis* via whole-genome next-generation sequencing (mNGS), the treatment regimen was promptly adjusted to a targeted approach centered on sulfamethoxazole-trimethoprim (SMZ-TMP 0.96 g tid) combined with linezolid (0.6 g q12h). SMZ-TMP, as the drug of choice for treating nocardiosis, demonstrated excellent tissue penetration and high susceptibility to *Nocardia* species in this case.

Recent systematic reviews indicate that SMZ-TMP retains high susceptibility to >90% of clinical *Nocardia* isolates, whereas linezolid is active against nearly all *Nocardia* species (susceptibility > 95%), and guidelines and clinical practice recommend it as a second-line or combination agent. The addition of linezolid enhanced the anti-gram-positive activity, and its distinctive pharmacokinetics further supported the control of disseminated systemic infection. When the pulmonary lesions progressed, amikacin (0.4 g qd) was added to establish a three-drug combination. Amikacin has been considered the most effective aminoglycoside in several multicenter studies, with *in vitro* susceptibility rates generally >90% against Nocardia9; thus, it is often used as an adjunct in severe or disseminated disease. When the patient developed myelosuppression during SMZ-TMP plus linezolid therapy, the medical team, adhering to the guidelines and literature, switched to amoxicillin/clavulanate monotherapy (9, 10). Amoxicillin/clavulanate remains active against certain *Nocardia* strains; surveillance data from Europe and East Asia suggest low MICs for some *N. brasiliensis* isolates (10), making it a reasonable alternative when first-line drugs are not tolerated.

#### 4.1.2 Management of *Enterococcus faecium* urinary tract infection

Regarding the management of *E. faecium* UTI, urine culture showed >105 CFU/mL, and susceptibility testing confirmed a multidrug-resistant strain, with susceptibility retained only to a few agents such as vancomycin and linezolid. Although linezolid has good activity against resistant enterococci, including vancomycin-resistant strains, its relatively low urinary concentrations limit its utility in UTIs (11, 12). Addressing this therapeutic dilemma highlights the complexity of clinical decision making. The combination of linezolid and SMZ-TMP leveraged pathogen-specific activities and potential synergy, thereby improving the overall therapeutic effect. This combined strategy offers a useful reference for similar mixed infections involving multidrug-resistant organisms.

#### 4.1.3 Management of drug-induced thrombocytopenia

Although the combination of linezolid and SMZ-TMP is effective for disseminated nocardiosis, the risk of myelosuppression cannot be overlooked. In this case, the platelet count decreased rapidly after 30 days of therapy (from 488 × 10^9^/L to 72 × 10^9^/L), accompanied by a decline in hemoglobin, which is typical of drug-related bone marrow suppression. To validate the causality of the adverse reaction, we applied the Naranjo Adverse Drug Reaction Probability Scale, which yielded a score of 7, indicating a “probable” relationship between the medications and thrombocytopenia. Previous studies have shown that linezolid can cause dose- and duration-dependent thrombocytopenia via the inhibition of mitochondrial ribosomal protein synthesis in precursor cells, leading to impaired megakaryocyte maturation and reduced platelet production (13). SMZ-TMP impairs hematopoiesis by inhibiting folate metabolism required for DNA synthesis in bone marrow cells (14). The concurrent use of these two agents likely exerted a synergistic suppressive effect on the bone marrow, resulting in severe hematopoietic inhibition. When combined, the risk is higher, with reported incidences of 30%–40% in long-term treatment populations (15). Therefore, many guidelines recommend at least twice-weekly complete blood counts during therapy and predefined intervention thresholds (e.g., PLT < 100 × 10^9^/L or a weekly decline >25%) to trigger drug discontinuation or regimen adjustment in a timely manner.

For adverse reaction management, this case adopted prompt discontinuation of linezolid and SMZ-TMP, instituted platelet-raising supportive measures, and switched the anti-nocardial regimen to amoxicillin/clavulanate, thereby preventing bleeding while maintaining treatment continuity. Studies suggest that alternative agents, such as minocycline or moxifloxacin, may be considered in high-risk patients (6), where feasible therapeutic drug monitoring (TDM) can be employed to mitigate toxicity risk. This case illustrates that the recognition and management of severe adverse reactions should follow the principles of early warning, rapid intervention, and individualized adjustment, ensuring safety while providing a referable approach for similar complex infections.

### 4.2 Continuous pharmaceutical care

In treating complex mixed infections, such as disseminated nocardiosis complicated by urinary tract infections, long-term, multi-drug combination regimens pose dual challenges to efficacy and safety. Against this backdrop, implementing systematic, continuous pharmaceutical monitoring is crucial for balancing the anti-infective efficacy with medication risks. Comprehensive, proactive pharmaceutical monitoring is an indispensable component for ensuring treatment success, spanning the entire lifecycle from “efficacy assessment – medication optimization – risk alerting – adherence management.” This is manifested in four key aspects:

Regarding dynamic efficacy assessment, pharmaceutical monitoring should evolve beyond singular clinical symptom observations to a multidimensional, integrated evaluation model incorporating clinical, biochemical, and imaging data. Beyond monitoring the resolution of skin abscesses and alleviation of respiratory/urinary symptoms, systematic tracking of inflammatory marker trends (e.g., CRP and PCT) and imaging progression (particularly chest CT) is essential. For disseminated nocardiosis, sequential imaging comparisons serve as a core basis for judging the lesion response and treatment stability. In this case, chest CT scans at days 30 and 60 confirmed radiological improvements, including reduced pulmonary nodules, thinned cavity walls, and absorbed inflammatory exudate, indicating the clear efficacy of antimicrobial therapy.

Regarding medication monitoring, pharmacists must ensure the standardized execution and dynamic optimization of treatment regimens. This process emphasizes precision medication management based on the physiological status of the patient. This includes adjusting drug dosages according to changes in renal and hepatic function (e.g., TMP-SMX), systematically assessing potential drug interaction risks (e.g., linezolid’s MAOI-like properties may enhance central nervous system drug effects; TMP-SMX may potentiate the hypoglycemic effects of sulfonylurea agents, necessitating enhanced blood glucose monitoring), and prudently evaluating the timing of transition from intravenous to oral sequential therapy based on infection control status and patient tolerance to maintain effective tissue drug exposure levels.

Regarding adverse reaction monitoring, this case utilized a structured pharmaceutical care model integrated with hospital information technology. Standardized and actionable monitoring and intervention protocols must be established to address the heightened risks associated with long-term, multi-drug regimens. Core measures include intensive blood count monitoring (recommended ≥2 times weekly) with a particular focus on platelet count changes. In this practice, the clinical pharmacist utilized an Electronic Medical Record (EMR) trigger tool to implement real-time monitoring. Clear intervention thresholds were pre-defined in the clinical protocol (e.g., PLT < 100 × 10^9^/L or a weekly decrease >25%) to enable the early identification and timely management of bone marrow suppression. Concurrently, routine monitoring of liver and kidney function, creatine kinase, and other indicators should be conducted alongside assessments of ototoxicity and nephrotoxicity risks for drugs, such as amikacin. Where feasible, therapeutic drug monitoring (TDM) can provide evidence for individualized dose adjustments of medications, such as linezolid, thereby ensuring efficacy while reducing toxicity risks (16).

Regarding adherence monitoring and patient education, the success of long-term therapy relies heavily on patient understanding and sustained cooperation with the treatment plan. Pharmacists can reduce medication errors and discontinuation risks through systematic in-person education during hospitalization and jointly developing clear discharge medication regimens and follow-up schedules (17). Simultaneously, establishing post-discharge follow-up mechanisms (e.g., telephone follow-ups or mobile platform management) enables the dynamic monitoring of medication adherence, adverse reactions, and disease progression while providing ongoing pharmaceutical guidance. When implementing outpatient parenteral antibiotic therapy (OPAT), pharmacist coordination throughout the treatment process significantly enhances treatment standardization and safety (18).

Ultimately, the systematic collection and analysis of medication information, adverse reaction events, and treatment outcomes from such complex infection cases enables the gradual development of institution-based pharmaceutical monitoring pathways and risk warning databases based on real-world data. This facilitates the transformation of pharmaceutical expertise from case-based practice to institutionalized, standardized management, demonstrating the professional value of clinical pharmacy in the precision treatment of infectious diseases.

### 4.3 Core value of pharmaceutical services and transferable experience

The successful diagnosis and treatment of disseminated *Nocardia brasiliensis* infection complicated by urinary tract infection in this case fundamentally demonstrate the synergistic effect achieved when pharmaceutical services are deeply integrated throughout the entire clinical decision-making process. From evaluating treatment strategies when initial empirical meropenem proved ineffective, to establishing “co-trimoxazole plus linezolid” as the core regimen based on mNGS results, to recommending amikacin augmentation for enhanced antimicrobial coverage during pulmonary lesion progression, and promptly switching to amoxicillin-clavulanate potassium upon severe thrombocytopenia— Pharmacists were fully involved in selecting, evaluating, and optimizing the antimicrobial regimen (19). Concurrently, high-frequency blood counts enabled the proactive monitoring of the risk of bone marrow suppression by linezolid combined with sulfamethoxazole. They also coordinated multidisciplinary collaboration among Infectious Diseases, Urology, and Radiology departments, guiding post-discharge adherence management for the patient (20). These interventions ensured the efficacy and continuity of antimicrobial therapy while successfully avoiding severe adverse drug reactions, highlighting the irreplaceable role of pharmaceutical services in personalized treatment of complex mixed infections.

Based on this case study, a universal pharmacovigilance pathway that is applicable to disseminated mixed infections across multiple systems can be summarized. In suspected disseminated infection scenarios: - Promote early implementation of rapid pathogen detection methods such as mNGS to facilitate pharmacist involvement in initial drug regimen design during empirical treatment (21); Establish a multidimensional monitoring system integrating “inflammatory markers—imaging studies—laboratory tests,” defining critical intervention thresholds such as platelet count <100 × 10^9^/L; dynamically evaluate treatment efficacy by correlating clinical manifestations with test results, promptly adjust antimicrobial regimens, and prospectively plan oral sequential therapy and post-discharge follow-up monitoring (22). Following validation in similar cases, this model holds promise for the development of hospital-wide pharmaceutical care guidelines for complex infections. The pharmacist-led adverse reaction early warning mechanism and multidisciplinary collaboration model also provide a replicable practice paradigm for standardized management of complex infections.

This study is a single-case report and has not yet systematically implemented therapeutic drug monitoring (TDM) or toxicity prediction analysis, resulting in certain limitations to its conclusions. Future research should integrate genomic resistance testing with pharmacokinetic/pharmacodynamic modeling to further optimize individualized treatment regimens. Additionally, for resource-constrained settings, risk-stratified pharmacotherapy monitoring strategies could be explored, moderately reducing the monitoring frequency during stable or low-risk disease phases to achieve a reasonable balance between healthcare quality and resource allocation.

## Data Availability

The original contributions presented in this study are included in this article/supplementary material, further inquiries can be directed to the corresponding author.

## References

[1] Lederman ER Crum NFA. Case series and focused review of nocardiosis: clinical and microbiologic aspects. *Medicine*. (2004) 83:300–13. 10.1097/01.md.0000141100.30871.39 15342974

[2] Brown-Elliott BA Brown JM Conville PS Wallace RJ. Clinical and laboratory features of the *Nocardia* spp. based on current molecular taxonomy. *Clin Microbiol Rev*. (2006) 19:259–82. 10.1128/CMR.19.2.259-282.2006 16614249 PMC1471991

[3] Lebeaux D Bergeron E Berthet J Djadi-Prat J Mouniée D Boiron P et al. Antibiotic susceptibility testing and species identification of *Nocardia* isolates: a retrospective analysis of data from a french expert laboratory, 2010-2015. *Clin Microbiol Infect*. (2019) 25:489–95. 10.1016/j.cmi.2018.06.013 29933049

[4] Kim S Shi HJ Jeon CH Kim SB Yi J Kim AR et al. Clinical characteristics, susceptibility profiles, and treatment of nocardiosis: a multicenter retrospective study in 2015-2021. *Int J Infect Dis*. (2023) 130:136–43. 10.3947/ic.2023.0032 36871785

[5] Brown-Elliott BA Biehle J Conville PS Cohen S Saubolle M Sussland D et al. Sulfonamide resistance in isolates of *Nocardia* spp. from a US multicenter survey. *J Clin Microbiol*. (2012) 50:670–2. 10.1128/JCM.06243-11 22170936 PMC3295118

[6] Uhde KB Pathak S McCullum I Jr. Jannat-Khah DP Shadomy SV Dykewicz CA et al. Antimicrobial-resistant *Nocardia* isolates, United States, 1995-2004. *Clin Infect Dis*. (2010) 51:1445–8. 10.1086/657399 21058914

[7] Wagenlehner FME Bjerklund Johansen TE Cai T Koves B Kranz J Pilatz A et al. Epidemiology, definition and treatment of complicated urinary tract infections. *Nat Rev Urol*. (2020) 17:586–600. 10.1038/s41585-020-0362-4 32843751

[8] Flores-Mireles AL Walker JN Caparon M Hultgren SJ. Urinary tract infections: epidemiology, mechanisms of infection and treatment options. *Nat Rev Microbiol*. (2015) 13:269–84. 10.1038/nrmicro3432 25853778 PMC4457377

[9] Restrepo A Clark NM. Infectious diseases community of practice of the American society of transplantation. *Nocardia* infections in solid organ transplantation: guidelines from the infectious diseases community of practice of the American society of transplantation. *Clin Transplant*. (2019) 33:e13509. 10.1111/ctr.13509 30817024

[10] Wang H Zhu Y Cui Q Wu W Li G Chen D et al. Epidemiology and antimicrobial resistance profiles of the *Nocardia* species in China, 2009 to 2021. *Microbiol Spectr*. (2022) 10:e0156021. 10.1128/spectrum.01560-21 35234511 PMC9045199

[11] Zhao M Liang L Ji L Chen D Zhang Y Zhu Y et al. Similar efficacy and safety of daptomycin versus linezolid for treatment of vancomycin-resistant enterococcal bloodstream infections: a meta-analysis. *Int J Antimicrob Agents*. (2016) 48:231–8. 10.1016/j.ijantimicag.2016.06.010 27475877

[12] Zhanel GG Love R Adam H Golden A Zelenitsky S Schweizer F et al. Tedizolid: a novel oxazolidinone with potent activity against multidrug-resistant gram-positive pathogens. *Drugs*. (2015) 75:253–70. 10.1007/s40265-015-0352-7 25673021

[13] De Vriese AS Van Coster R Smet J Seneca S Lovering A Van Haute LL et al. Linezolid-induced inhibition of mitochondrial protein synthesis. *Clin Infect Dis.* (2006) 42:1111–7. 10.1086/501356 16575728

[14] Ho JMW Juurlink DN. Considerations when prescribing trimethoprim–sulfamethoxazole. *CMAJ*. (2011) 183:1851–8. 10.1503/cmaj.111152 21989472 PMC3216436

[15] Natsumoto B Yokota K Omata F Furukawa K. Risk factors for linezolid-associated thrombocytopenia in adult patients. *Infection*. (2014) 42:1007–12. 10.1007/s15010-014-0674-5 25119433 PMC4226929

[16] Kirkpatrick ER Hand EO Hughes DW. Impact of implementing pharmacist review and monitoring of outpatient parenteral antimicrobial therapy. *Open Forum Infect Dis*. (2020) 7:S365. 10.1177/0897190014544786 25107418

[17] Rivera CG Mehta M Ryan KL Stevens RW Tucker KJ Mahoney MV. Role of infectious diseases pharmacists in outpatient intravenous and complex oral antimicrobial therapy: society of infectious diseases pharmacists insights. *J Am Coll Clin Pharm*. (2021) 4:1161–9. 10.1002/jac5.1473

[18] Stashluk S Ramos M Carettini T Cutrell JB Mathew S Monogue M et al. Bridging the gap: opportunities for transitions of care pharmacist review of outpatient parenteral antimicrobial therapy prescriptions prior to hospital discharge. *Antimicrob Stewardship Healthc Epidemiol.* (2024) 4:e50. 10.1017/ash.2024.52 38655020 PMC11036442

[19] Epperson TM Bennett KK Kupiec KK Speigel K Neely SB Resman-Targoff BH et al. Impact of a pharmacist-managed outpatient parenteral antimicrobial therapy (OPAT) service on cost savings and clinical outcomes at an academic medical center. *Antimicrob Stewardship Healthc Epidemiol.* (2023) 3:e15. 10.1017/ash.2022.374 36714295 PMC9879875

[20] Manders IG Comello D Souverein D Euser S Herpers BL Vetten J et al. The impact of a structured outpatient parenteral antimicrobial therapy (OPAT) programme on quality of care, optimisation of antimicrobial use, and healthcare costs: a retrospective cohort study. *Antibiotics*. (2025) 14:1103. 10.3390/antibiotics14111103 41301598 PMC12649236

[21] Nampoothiri V Hisham M Mbamalu O Mohamed ZU Singh SK Charani E. Evolution of pharmacist roles in antimicrobial stewardship: a 20-year systematic review. *Int J Infect Dis*. (2025) 151:107306. 10.1016/j.ijid.2024.107306 39551088

[22] Durojaiye OC Fiori C Cartwright K. Delivery of outpatient parenteral antimicrobial therapy (OPAT) in an ever-changing national health service (UK): benefits, barriers, and opportunities. *Antibiotics*. (2025) 14:451. 10.3390/antibiotics14050451 40426518 PMC12108282

